# In the face of threat: neural and endocrine correlates of impaired facial emotion recognition in cocaine dependence

**DOI:** 10.1038/tp.2015.58

**Published:** 2015-05-26

**Authors:** K D Ersche, C C Hagan, D G Smith, P S Jones, A J Calder, G B Williams

**Affiliations:** 1 Department of Psychiatry, University of Cambridge, Cambridge, UK; 2 Behavioural and Clinical Neuroscience Institute, University of Cambridge, Cambridge, UK; 3 Department of Psychology, University of Cambridge, Cambridge, UK; 4 Medical Research Council Cognition and Brain Sciences Unit, Cambridge, UK; 5 Wolfson Brain Imaging Centre, University of Cambridge, Cambridge, UK

## Abstract

The ability to recognize facial expressions of emotion in others is a cornerstone of human interaction. Selective impairments in the recognition of facial expressions of fear have frequently been reported in chronic cocaine users, but the nature of these impairments remains poorly understood. We used the multivariate method of partial least squares and structural magnetic resonance imaging to identify gray matter brain networks that underlie facial affect processing in both cocaine-dependent (*n*=29) and healthy male volunteers (*n*=29). We hypothesized that disruptions in neuroendocrine function in cocaine-dependent individuals would explain their impairments in fear recognition by modulating the relationship with the underlying gray matter networks. We found that cocaine-dependent individuals not only exhibited significant impairments in the recognition of fear, but also for facial expressions of anger. Although recognition accuracy of threatening expressions co-varied in all participants with distinctive gray matter networks implicated in fear and anger processing, in cocaine users it was less well predicted by these networks than in controls. The weaker brain-behavior relationships for threat processing were also mediated by distinctly different factors. Fear recognition impairments were influenced by variations in intelligence levels, whereas anger recognition impairments were associated with comorbid opiate dependence and related reduction in testosterone levels. We also observed an inverse relationship between testosterone levels and the duration of crack and opiate use. Our data provide novel insight into the neurobiological basis of abnormal threat processing in cocaine dependence, which may shed light on new opportunities facilitating the psychosocial integration of these patients.

## Introduction

Facial expressions of emotion are important social signals of information, reflecting the emotional state of others and helping us to predict their behavior and adjust our responses accordingly.^1^ Chronic abuse of stimulant drugs like cocaine and amphetamines has repeatedly been shown to selectively impair the recognition of fear in others^2, 3, 4^—an important social cue that signals immediate threat^5^—and this failure can lead to maladaptive behavior and social difficulties.^6^ Yet the relationship between stimulant drug dependence, impairments in fear recognition and social behavior remains elusive. It is conceivable that these impairments may predate stimulant dependence as adolescents with conduct disorder—who have an increased risk for developing drug addiction in adulthood^7^—exhibit impairments in recognizing negative facial affect, including fear.^8, 9^ An alternative, but not mutually exclusive, possibility is that prolonged drug abuse impairs emotion processing abilities. However, prior studies failed to find a direct relationship between the duration of stimulant use and fear recognition impairments,^2, 4, 10^ suggesting that these relationships are more likely to be indirect.

Imbalance in the levels of steroidal hormones, such as cortisol and testosterone, may have a mediating role. Cortisol and testosterone are the end products of the hypothalamus–pituitary–adrenal (HPA) axis and the hypothalamus–pituitary–gonadal (HPG) axis, respectively, and together they regulate an individual's response to threat.^11^ Cortisol directly influences autonomic neural systems associated with the processing of threatening information, such as those conveyed by fearful or angry facial expressions,^12, 13, 14^ while testosterone is thought to modulate the behavioral response to threat.^15, 16^ Both hormones have mutually antagonistic actions—that is, cortisol attenuates the production of testosterone by suppressing HPG-activity, and testosterone reduces cortisol by inhibiting HPA-activity at the level of the hypothalamus.^17^ An imbalance between the HPA and HPG axes, as measured by peripheral levels of cortisol and testosterone, has been shown to mediate facial affect processing through their endocrine actions on neural activity in the amygdala and orbitofrontal cortex.^18, 19, 20, 21, 22, 23^ These areas, along with the inferior occipital and fusiform gyri and the prefrontal cortex, make up the distributed network of cortical and subcortical brain structures that process facial affect.^24^ Thus, as both hormones can affect brain structure^25, 26^ and functional connectivity during facial emotion recognition,^27, 28^ it is conceivable that hormonal changes mediate threat processing through changes in these underlying structural brain networks. Indeed, decreased functional connectivity or damage to structures within this network have repeatedly been linked with impairments in emotion perception, including fear recognition.^29, 30, 31^

Preclinical evidence suggests that repeated use of cocaine enhances HPA axis activity and suppresses the HPG axis,^32^ as reflected by increased cortisol and decreased testosterone levels.^33, 34, 35^ However, studies of hormonal changes in human long-term cocaine users have been inconsistent.^36, 37^ Using data-driven multivariate analysis methods, we sought to first determine the neural correlates associated with facial threat recognition, and subsequently investigate the influence of steroidal hormones on this brain-behavior relationship. We hypothesized that imbalances between HPA and HPG axes activity in cocaine-dependent individuals (CDIs) would explain impairments in recognizing fear via associated neuroanatomical networks.

## Materials and methods

### Study sample

Sixty-five male volunteers, aged between 20 and 60 years, were recruited from the local community. Thirty-five individuals had a history of chronic cocaine abuse, satisfying the DSM-IV-TR^38^ criteria for cocaine dependence, whereas the remaining 30 individuals were healthy and without a personal and family history of drug abuse. Exclusion criteria for all volunteers included a lifetime history of a psychotic disorder, neurological illness or traumatic head injury, an autoimmune or metabolic disorder, or HIV-infection. All volunteers consented in writing before being screened for current psychiatric disorders using the Mini-International Neuropsychiatric Inventory.^39^ Psychopathology in drug users was further evaluated using the Structured Clinical Interview for DSM-IV.^40^ All participants completed the National Adult Reading Test^41^ to provide an estimate of verbal IQ, the State–Trait Anxiety Inventory^42^ to record temporary and stable feelings of anxiety, and the Beck Depression Inventory (BDI-II)^43^ to measure depressive mood. Before behavioral tasks, a blood sample was drawn in all participants to measure serum levels of cortisol and testosterone. Urine samples were tested for undeclared drugs; all samples provided by the healthy volunteers were negative and all except four urine samples provided by CDIs tested positive for stimulants. The protocol was approved by the National Research Ethics Committee (NREC10/H0306/69, PI: KD Ersche); data from both groups have been published previously.^44, 45^

All CDIs were non-treatment seeking and had been actively using cocaine/crack-cocaine for an average of 17 years (±8.0 s.d.). Half of the CDIs smoked (*n*=12) or injected (*n*=9) crack-cocaine, while the other half used powdered cocaine intranasally (*n*=14). Besides meeting the DSM-IV-TR criteria for cocaine dependence, several CDIs also met criteria for dependence on another substance (91% nicotine, 43% opiates, 29% alcohol, 20% cannabis, 3% amphetamines) and used other drugs sporadically (68% cannabis, 20% sedatives, 15% opiates, 14% ecstasy, 3% hallucinogens). Eleven CDIs were prescribed methadone (mean dose: 55 mg±16.2 s.d.), three were prescribed buprenorphine (mean dose: 3 mg±2.6 s.d.) and four received narcotic-like pain relief medication on prescription. Three CDIs were prescribed antidepressants, three were prescribed benzodiazepines and one received d-amphetamines on prescription. None of the healthy volunteers satisfied criteria for alcohol abuse or dependence, nor were they taking prescribed or illicit drugs on a regular basis. Seventy percent of healthy volunteers were either past or current smokers of tobacco, and 57% reported having social experiences with cannabis.

### Behavioral, brain and endocrine data collection

All participants first completed the Benton Facial Recognition Test,^46^ a face-matching task used to verify the ability to perceptually discriminate faces. Participants were presented with six black-and-white photographs of unfamiliar faces and asked to identify first one and subsequently three photographs that matched the target face simultaneously shown. A performance score was calculated from the number of correct responses, taking into account participants' age and education. Next, participants were tested on facial expression recognition using the computerized Emotion Hexagon task.^47^ It consisted of morphed black-and-white images spanning six facial expression pairs: happiness–surprise, surprise–fear, fear–sadness, sadness–disgust, disgust–anger, anger–happiness. The ratio of blending within each expression pair varied in 20% increments (that is, 90% happy blended with 10% surprise, 70% happy and 30% surprise, 50% happy and 50% surprise, 30% happy and 70% surprise and 10% happy blended with 90% surprise). Faces were shown for a maximum of 5 s, and participants were asked to judge by button-press which of the six emotions each face was expressing. Participants had no time limit for their judgments and received no feedback about the accuracy of their responses. Task performance was reflected by the percentage of correct responses for each emotion.

After completion of the behavioral tasks (between 1200 and 1300 hours GMT), blood samples were drawn from each participant and sent to the Department of Pathology at Addenbrooke's Hospital (www.cuh.org.uk), where serum levels of cortisol and testosterone were determined using competitive chemiluminescent immunoassays on the Siemens ADVIA Centaur Immunoassay System. Participants then underwent a whole-brain T1-weighted magnetic resonance (MR) scan at the Wolfson Brain Imaging Centre, University of Cambridge, UK. The MR images were acquired using a Siemens TIM-Trio 3T system with a magnetization-prepared rapid acquisition gradient-echo sequence (176 slices of 1mm thickness, TR=2300 ms, TE=2.98 ms, TI=900 ms, flip angle=9°, FOV= 240 × 256). Out of the final sample of 58 participants, three MR imaging scans (two controls, one CDI) were of poor quality and therefore not included in the analysis.

### Statistical analyses

Three participants' behavioral data were lost due to technical failure (one control, two CDIs). CDIs without a stimulant-positive urine screen (*n*=4) were also excluded to avoid potentially confounding effects of drug abstinence, leaving a final sample of 58 participants (29 individuals per group). Data were analyzed using the Statistical Package for the Social Sciences (SPSS, version 22; IBM, Armonk, NY, USA). Group differences regarding demographic, behavioral and endocrine measures were analyzed using independent samples *t*-tests. Performance on the Hexagon task was analyzed using multivariate analysis of variance to take into account the relationships between the six emotions. We initially included verbal IQ, depressive mood and trait-anxiety as covariates in the analysis to statistically control for group differences in these variables, but all variables interacted with group status, suggesting that they are defining features of these groups. Consequently, co-varying for them would not be appropriate.^48^ To determine potentially confounding effects of the aforementioned demographic variables and the influence that comorbidity had on the results, we performed a two-step hierarchical regression model. In step 1, we included dependence status as three separate dichotomous variables (D1: not cocaine-dependent, cocaine-dependent; D2: not opiate-dependent, opiate-dependent; D3: not alcohol-dependent, alcohol-dependent) and then added the continuous variables verbal IQ, depressive mood and trait-anxiety to the model in step 2. Significant results of the regression model were tested for mediator effects using the process software tool (version 2.13) by Andrew F. Hayes (www.afhayes.com), which was implemented in SPSS. We also calculated Pearson's correlation coefficients to evaluate putative relationships between outcome variables and drug-taking measures. All statistical tests were two-tailed and significance levels were set at 0.05.

Gray matter density maps were analyzed using FSL-VBM (www.fmrib.ox.ac.uk/fsl/fslvbm, v.4.1). Non-brain tissues were removed using the brain extraction tool of FSL and tissue-type segmentation was performed using FAST. The resulting gray matter partial-volume images were aligned to MNI-152 standard space using the affine registration tool FLIRT, followed by nonlinear registration using FNIRT, which uses a *b*-spline representation of the registration warp field. A study-specific gray matter template was made to which the native gray matter images were nonlinearly re-registered. To correct for local expansion or contraction, the registered partial-volume images were modulated by division with the Jacobian warp field. The modulated segmented images were then smoothed with an isotropic Gaussian kernel with full width half maximum=2.3 mm. Group comparisons of the gray matter maps were performed using CamBA software for permutation testing,^49^ v.2.3.0 (http://www.bmu.psychiatry.cam.ac.uk/software/) and thresholded at cluster-level statistics of *η*=1 error clusters per image.

### The influence of steroidal hormones in predicting facial affect processing from brain structure

We used partial least squares methods^50^ to determine covariance between brain regions (voxels) across the entire brain and successful task performance (recognition accuracy). For the calculation of the correlation maps, we only included brain regions where the mean gray matter density over all participants was >0.01. We determined the overall strength of each correlation map by calculating the root-mean-squared correlation coefficient over the entire brain.^51^ The significance of the correlation pattern was then tested using permutation methods—that is, the input images were randomly re-labeled and the root-mean-squared correlation was re-calculated to generate a null distribution for the hypothesis (1,000 permutations were used).

In partial least squares method, each voxel in the correlation map is given a numerical weight (salience) reflecting how strongly that voxel is related to the behavioral variable of interest. Salience can be either positive or negative depending on whether the voxel shows a positive or negative relationship with the pattern identified. Partial least squares method also computes a brain score that reflects the strength of each participant's brain-behavior relationship. As CDIs exhibited recognition impairments for both fearful and angry facial expressions, we investigated both expressions separately. Brain scores were imported into SPSS for statistical group comparisons,^51^ and multiple stepwise regression was used to determine the influence of testosterone and cortisol levels on the brain scores for fear and anger recognition. We then conducted bootstrap-mediation analysis restricted to the significant results of the regression analysis using the process software tool (version 2.13; www.afhayes.com) implemented in SPSS.

## Results

### Demographic and clinical data

Descriptive data is shown in Table 1. The two groups were well matched for age but differed significantly with regard to verbal IQ, anxiety and dysphoric mood. Serum levels of testosterone were significantly reduced in CDIs, while cortisol levels were not measurably different between the groups. CDIs and healthy control volunteers did not differ on vital signs, including pulse rate (*t*_56_=1.12, *P*=0.247), systolic (*t*_56_=0.12, *P*=0.908) and diastolic blood pressure (*t*_56_=0.98, *P*=0.333), indicating that the CDIs were not acutely intoxicated.

No differences were observed between subgroups of CDIs with comorbid opiate or alcohol dependence regarding demographics, intelligence, anxiety, mood, physiological measures and duration or compulsive pattern of stimulant use (all *P*>0.1). However, significantly lower levels of testosterone were found in CDIs with comorbid opiate dependence compared with their non-opiate-dependent counterparts (*t*_27_=−2.36, *P*=0.026), suggesting that the low levels of testosterone observed in the cocaine group may have been driven, at least in part, by the low testosterone levels in CDIs with comorbid opiate dependence.

### Behavioral data: facial identification and affect processing

Compared with their healthy peers, CDIs performed equally well in matching the unfamiliar faces (*t*_54_=0.10, *P*=0.324), confirming no significant group differences in facial discrimination ability. However, one participant in each group (<4% of the entire sample) scored outside the normal range; this is in keeping with predictions that ~8% of normal individuals without a known pathology underperform on this test.^52^ To ensure that our findings were not confounded by deficits in facial perception, all analyses were repeated after removal of these two individuals.

**Figure 1:**
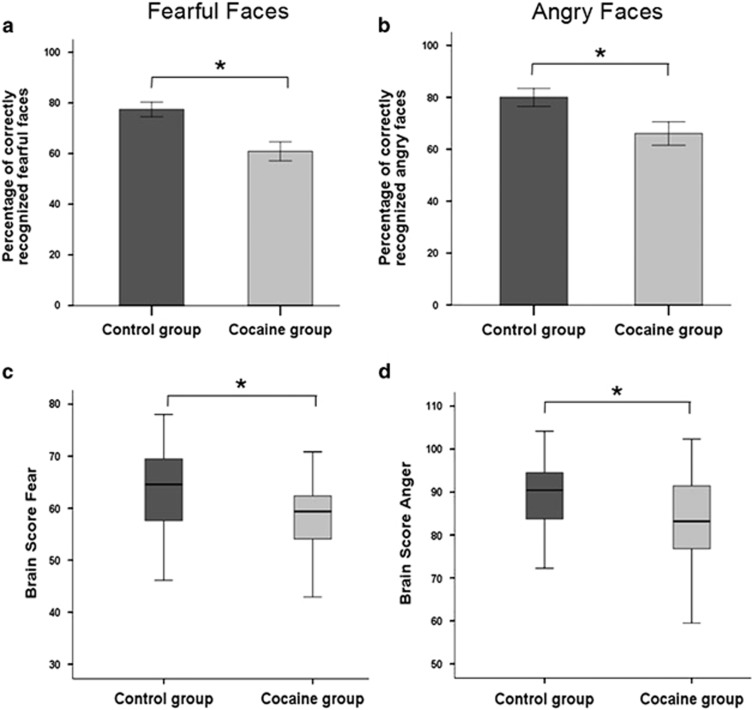
Group comparisons of facial affect recognition performance. As shown in the two graphs at the top, CDIs recognized significantly fewer facial expressions depicting fear (**a**) and anger (**b**) compared with their non-drug-using healthy peers. To identify the neural correlates of fear and anger recognition impairments in the cocaine group, we used PLS analysis to identify gray matter networks that co-vary with participants' recognition performance of fearful and angry faces, respectively. PLS determines covariance between brain voxels and recognition accuracy across the entire brain and computes from the summary of all the voxels of the network for each participant a brain score, which indicates how well the identified network reflects behavioral performance. The two graphs at the bottom show that CDIs' brain scores for both fear (**c**) and anger (**d**) were significantly lower compared with those of their healthy peers, indicating that CDIs ability to recognize fearful and angry faces is less well explained by the identified gray matter networks. CDI, cocaine-dependent individual; PLS, partial least squares method.

As shown in Figure 1, CDIs exhibited deficits on the Emotion Hexagon task in recognizing not only fearful (F_1,56_=12.34, *P*=0.001) but also angry (F_1,56_=6.03, *P*=0.017) facial expressions. On closer inspection, we noticed that CDIs misclassified expressions of fear significantly more often than control volunteers as expressions of either sadness (Fisher's exact *P*=0.032) or surprise (Fisher's exact *P*=0.070). No systematic misclassifications were observed for angry faces. The two groups did not differ with respect to time needed for classifications (Table 1).

Hierarchical regression revealed that drug dependency (*R*^2^=0.25, F_3,51_=5.72, *P*=0.002) accounted for a quarter of the variance of fear recognition (Δ*R*^2^=0.25, *P*=0.002), and the inclusion of demographic variables to the model (*R*^2^=0.38, F_6,48_=4.92, *P*=0.001) explained an additional 13% of the variance (Δ*R*^2^=0.13, *P*=0.027). As can be seen from Table 2, cocaine dependence was significant at step 1 (*β*=−0.37; *P*=0.023), but this effect did not survive when verbal IQ (*β*=0.42; *P*=0.005) was included in the model at step 2, suggesting that the significant direct effect of cocaine dependence was mediated by verbal IQ. Drug-dependency also had a significant effect on the recognition of anger (*R*^2^=0.34, F_3,51_=10.37, *P*<0.001), explaining 38% of the variance (Δ*R*^2^=0.38, *P*<0.001). When demographic variables were added to the model (*R*^2^=0.43, F_6,48_=7.66, *P*<0.001), they explained an additional 11% of the variance (Δ*R*^2^=0.11, *P*=0.024). As shown in Table 2, the significant effect of dependency was largely driven by opiate dependence (*β*=−0.60; *P*<0.001) and this effect survived when verbal IQ (*β*=−0.35; *P*=0.009) was included in the model, suggesting that opiate dependence had a critical role in the observed impairments in anger recognition. Indeed, when opiate-dependent patients were excluded from the sample, the groups no longer differed in terms of anger recognition (F_1,44_=0.03, *P*=0.876) but remained significantly different for fear recognition (F_1,44_=5.58, *P*=0.023).

### Brain data and prediction of individual variation in facial affect processing

**Figure 2:**
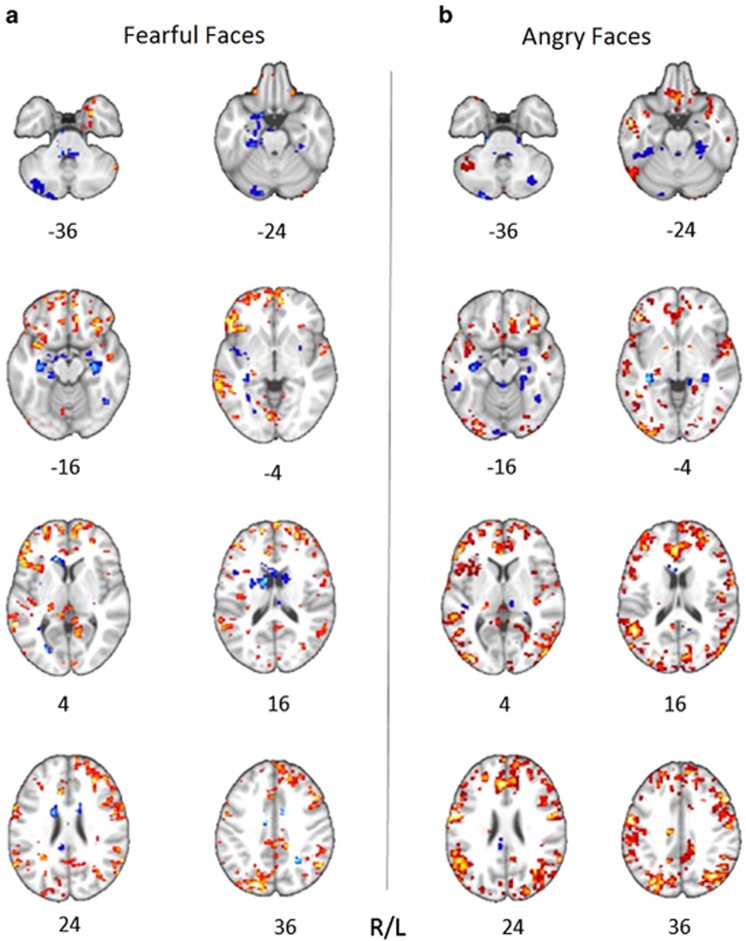
Clusters of gray matter density that are associated with successful recognition of fearful (**a**) and angry (**b**) facial expressions, as identified by PLS. The numerical values within each cluster are known as *salience* (comparable with the component load in a principal component analysis), reflecting the direction of the relationship between gray matter density and behavior. Regions colored in red/yellow indicate positive salience: increased gray matter density was associated with poor recognition performance (that is, low level of accuracy). Regions colored in blue reflect negative salience: increased gray matter density was associated with good recognition performance (that is, high level of accuracy). The numbers below the brain slice denote the z-dimension of each slice in Montreal Neurological Institute (MNI) space. Each image was thresholded at *Z*>1.96. PLS, partial least squares method; R/L, right/left.

Univariate comparisons of whole-brain gray matter maps did not reveal any significant differences between the groups. Permutation tests showed that networks of gray matter variation significantly correlated in both groups with recognition of fear (*P=*0.035) and anger (*P<*0.001). The networks for fear and anger recognition both encompassed distributed clusters across both hemispheres in the cingulum, inferior orbitofrontal gyrus, middle frontal gyrus, middle and superior temporal cortices, and fusiform gyrus, as well as parietal regions and subcortical structures including the amygdala and the hippocampus (Figure 2; for anatomical details, see Supplementary Tables S1 and S2). No significant overall laterality effects were identified for either the cluster size or the significant salience within each region for either fear or anger. The two networks did, however, differ notably in terms of cluster size and salience, reflecting the degree of regional brain-behavior correlation for each expression. For example, deficits in gray matter volume in the amygdala correlated with performance for both facial expressions, but the cluster size for fear was smaller and with higher salience than for anger, whereas the cluster for anger recognition comprised a larger volume but had lower salience. Group comparisons of the brain scores, which reflect the degree to which an individual's gray matter distribution explains their task performance, showed significantly lower brain scores in CDIs for both fear (*t*_53_=2.88, *P*=0.006) and anger (*t*_53_=2.04, *P*=0.046) compared with control volunteers (Figure 1).

**Figure 3:**
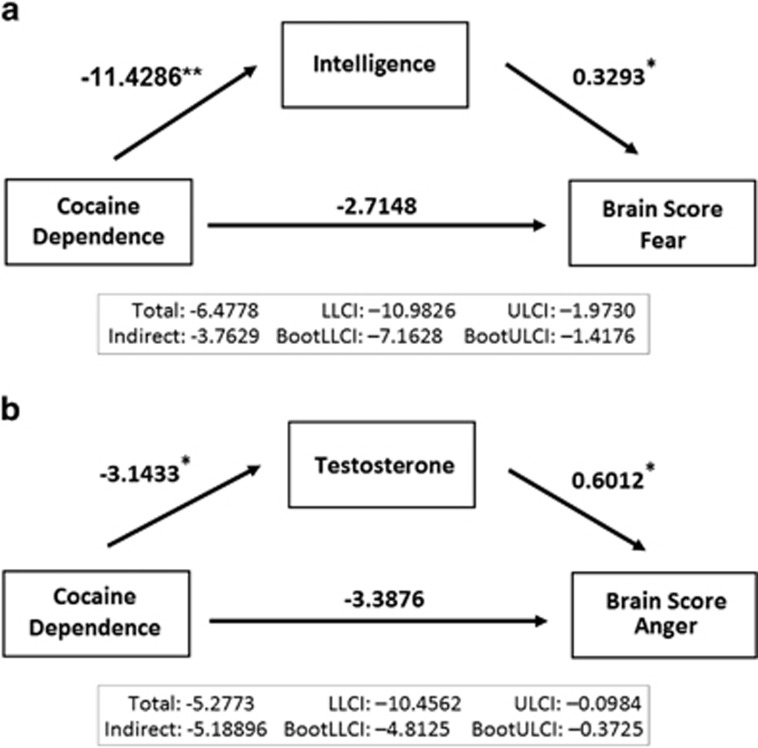
Unstandardized regression coefficients and bias-corrected 95% CI for the indirect effect from a bootstrap-mediation analysis that found that (**a**) intelligence mediated the relationship between cocaine dependence and the brain-behavior network implicated in fear recognition and (**b**) testosterone levels mediated the relationship between cocaine dependence and the brain-behavior network implicated in anger recognition. CI, confidence interval. *denotes significance at *P*<0.05. **denotes significance at *P*<0.001.

Hierarchical regression for the brain score of fear revealed a significant effect of drug dependency (*R*^2^=0.20, F_3,50_=5.52, *P*=0.002), explaining a quarter of the variance (Δ*R*^2^=0.25, *P*=0.002). Inclusion of demographic variables to the model (*R*^2^=0.29, F_6,47_=4.63, *P*=0.001) accounted for a further 12% of the variance (Δ*R*^2^=0.12, *P=*0.037). Although cocaine dependence significantly predicted the brain score of fear (*β*=−0.35, *P*=0.031), this effect did not survive when verbal IQ (*β*=0.35,*P*=0.018) was added to the model in step 2 (see Table 2), suggesting a mediating effect of IQ. We subsequently confirmed this in a mediation analysis showing that the significant direct effect of cocaine dependence on the fear brain score (*t*=−2.89, *P*=0.006) became nonsignificant (*t*=−1.03, *P*=0.306) when IQ (*t*=2.48, *P*=0.016) was included in the model (see Figure 3a).

With regard to the brain score of anger, there was also a significant effect of dependence (*R*^2^=0.31, F_3,50_=7.30, *P*<0.001), which accounted for 30% of the variance (Δ*R*^2^=0.31, *P<*0.001). As shown in Table 2, opiate dependence significantly predicted the brain score of anger (*β*=−0.516, *P*=0.001), and the inclusion of demographic variables (*R*^2^=0.37, F_6,47_=4.60, *P*=0.001) did not explain any further variance (Δ*R*^2^=0.07, *P*=0.196).

### Prediction of individual variation in brain-behavior networks by steroidal hormones

We used multiple stepwise regression analysis to further examine the extent to which steroidal hormone levels accounted for participants' brain-behavior relationships. The model revealed that testosterone accounted for 12% of the variance in brain scores associated with angry facial expression recognition (*R*^2^=0.13, F_1,52_=8.53, *P*=0.008); cortisol had no explanatory value. We subsequently discovered significant group-by-testosterone interactions on variations in the anger brain score (F_2,52_=4.41, *P*=0.017). A mediation analysis confirmed that the direct effect of cocaine on testosterone was significant (*t*=−2.29, *P*=0.026), as were the direct effects of both testosterone (*t*=2.44*, P*=0.018) and cocaine dependence (*t*=−2.04, *P*=0.048) on the anger brain score, respectively. However, when testosterone was added to the model, the direct effect of cocaine dependence was no longer significant (*t*=−1.31, *P*=0.196; see Figure 3b), suggesting that testosterone is mediating the effect of cocaine on the anger brain score. For fear recognition, neither cortisol nor testosterone levels accounted for variations in brain score.

### Relationships with drug use and physiological indices

Brain scores related to the recognition of angry faces in CDIs were significantly inversely correlated with duration of crack use (*r*=−0.44, *P*<0.05), but not with duration of powdered cocaine or opiate use (both *P*>0.5). Testosterone levels were also inversely correlated with the duration of crack (*r*=−0.51, *P*=0.012) and opiate use (*r*=−0.57, *P*=0.021), but not with the use of powdered cocaine (*r*=−0.17, *P*=0.409). No relationships were identified with regard to fearful face recognition brain score or cortisol levels.

## Discussion

Collectively, our data provide novel insight into the neurobiological basis of abnormal threat processing in cocaine dependence. As hypothesized, and consistent with previous studies,^2, 3, 10, 53^ we show that cocaine dependence is associated with impaired recognition of fearful facial expressions and may also extend to another threat-related expression—anger. In contrast to previous research, we reveal the important influence of intelligence on facial threat recognition and the related variations in anatomical brain structure. Although both groups relied upon the same gray matter networks, CDIs' recognition accuracy was less well predicted by these networks than in controls. This weaker brain-behavior relationship for fear recognition in CDIs was largely explained by group differences in intelligence levels. As shown in Figure 3, low IQ appeared to mediate fear recognition performance in CDIs. By contrast, impairments in anger recognition were mediated by the significantly reduced levels of testosterone in CDIs.

### Facial fear recognition

Out of the six basic emotions, fear is consistently the most difficult facial expression to recognize,^54^ and variations in recognition accuracy have frequently been linked with indices of intelligence.^55, 56, 57^ Intelligence also seems to be particularly important for individuals who have to compensate for dysfunctional brain networks or damaged brain tissue.^58^ Further, when psychiatric patients are compared with IQ-matched control volunteers on tests of emotion processing, their performance is not measurably impaired despite persisting perturbations in underlying functional neural networks.^59, 60, 61^ However, prior research in stimulant drug users has not investigated the influences of intelligence on fear recognition performance.^2, 3, 4^ Therefore, we used the National Adult Reading Test to estimate pre-morbid (not current) intellectual ability,^62^ revealing a potential relationship between IQ and fear recognition in CDIs. Given that stimulant drugs have been shown to enhance facial emotion recognition,^63^ it is tempting to speculate whether fear recognition impairments might impact the risk of cocaine abuse in individuals with low IQ. The relationship between intelligence and cocaine use is generally an interesting one: high IQ has been associated with an increased likelihood of experimenting with illicit drugs, including cocaine,^64^ while the risk of developing dependence in individuals with low IQ is thought to be mediated by conduct problems during childhood.^65^ One may speculate whether the impairments in fear recognition frequently reported in adolescents with conduct disorder might influence their increased risk of developing drug dependence.

In light of the strong genetic contribution to both intelligence and brain morphology,^66, 67^ it is unsurprising that disruptions in brain networks during emotion recognition have been reported not only in psychiatric patients, but also in their unaffected first-degree relatives.^68, 69^ Shared variance between family members in brain-behavior networks implicated in fear processing is particularly interesting in light of the social context in which fear is learned,^70^ as childhood adversity has been shown to either improve^71, 72^ or impair fear recognition performance.^73^ Comparisons between CDIs and their siblings who share adverse childhood experiences^74^ may thus provide insight into compensatory mechanisms in fear processing in those siblings who do not abuse drugs, possibly elucidating sources of resilience.

Finally, attention should be drawn to two observations that may, at first glance, appear counter-intuitive. First, variations in trait-anxiety did not affect fear recognition performance in either group. Prior research suggests that anxious individuals exhibit hypervigilance to facial signals of covert fear,^75, 76^ but in our study fearful faces were shown overtly for an extended period of time, and as such these observations may not be comparable. Second, although the HPA axis is involved in fear processing, cortisol levels and fear recognition performance were unrelated in both groups. Indeed, although acute changes in cortisol have been linked with processing threat-related stimuli,^12, 77^ basal levels seem to be unrelated to fearful recognition accuracy, except in individuals with increased risk for recurrent depression.^78^

### Facial anger recognition

In the current study, emotion recognition impairments in CDIs also extended to expressions of anger. Converging lines of evidence from clinical and experimental research indicate that striatal dopaminergic transmission modulates recognition accuracy of angry faces.^79, 80, 81^ However, although striatal dopamine function is diminished in cocaine dependence,^82^ impairments in anger recognition have rarely been reported previously in CDIs, except in those who are co-dependent on a variety of other drugs.^53, 83^ Additional factors are thus likely to mediate the processing of angry faces. Testosterone levels have been suggested to interact with striatal dopamine function,^84^ possibly by modulating neural reactivity to facial threat,^18, 19^ thereby mediating individuals' attention and behavioral reactions to angry expressions.^85^ Indeed, mediation analysis confirmed that low testosterone levels in CDIs largely explained their poor recognition of angry faces and the weak relationship between recognition performance and underlying gray matter networks (Figure 3).

It is of note that testosterone levels were significantly reduced in CDIs with comorbid opiate dependence, possibly due to the inhibitory effects of opiates on gonadal function.^86^ Regression analysis further confirmed the significant effect of comorbid opiate dependence on anger recognition, suggesting that the low testosterone levels associated with opiate dependence drive the impairments in anger recognition. This raises the question as to why impairments in angry facial expressions have previously not been reported in opiate-dependent individuals without concomitant crack-cocaine use.^87^ Possibly, the observed impairments in angry face perception reflect a combined effect of chronic cocaine and opiate use on HPA and HPG axes hormones,^88^ potentially mediated by altered vasopressin secretion in the hypothalamus^89^ and thereby influencing social behavior.^90, 91^ Given that neuropeptides like oxytocin and vasopressin are being discussed as potential targets for treatment of drug addiction,^92, 93^ facial affect recognition tasks could be useful for predicting social implications associated with this treatment in this highly prevalent but difficult-to-treat subgroup of crack-cocaine and heroin-dependent individuals.^94^

Angry faces are an important social sign of disapproval^95^ and may, therefore, be perceived as threatening,^96^ triggering avoidance responses in anxious individuals or aggression in dominant, status-seeking individuals.^15^ Failure to recognize angry faces thus has dramatic implications for social behavior,^97^ with a potential subsequent impact on rehabilitation in CDIs.^98^ The ratio between testosterone and cortisol levels seems to determine individuals' responses to confrontation,^99^ with an increased testosterone–cortisol ratio associated with reactive aggression.^16, 100^ As stated above, in our sample, CDIs' testosterone levels were significantly reduced relative to cortisol, suggesting a socially avoidant response tendency,^90, 101^ an effect previously reported in animal models of cocaine dependence.^102^

### Methodological considerations and outlook

Although our findings shed new light on disruptions associated with emotion processing in cocaine dependence, they also raise a number of questions that warrant further investigation. The group difference in National Adult Reading Test scores and the restriction of threat cues to facial expressions should be considered as a limitation to the study; specifically, the latter precludes inferences as to whether the impairments seen extend to other threat signals. The role of gender also warrants clarification as the current study only included male volunteers. Further, our study does not address the question about the sequelae of threat recognition impairments in CDIs during addiction recovery. Future studies may, therefore, consider determining whether the observed impairments are reversed following prolonged drug abstinence.

The multivariate nature of partial least squares provides the closest structural analog to functional connectivity studies, which has allowed us to relate the complex neuropathology of addiction to recognition performance in the same manner as the associated functional networks of threat processing that have previously been identified.^24^ Functional pharmacological and endocrine challenge studies are now needed to identify the mechanisms underpinning the described disruptions in threat processing. Relationships between recognition performance and brain structure, as we have shown here, may open up new avenues for elucidating the nature of abnormal functional brain activity previously reported in individuals at risk for addition^69^ and with particularly high recognition accuracy.^103^

## Figures and Tables

**Table 1:** Demographics, clinical variables and facial affect recognition performance for healthy male volunteers and cocaine-dependent men

*Descriptive variables*	*Control group (*N=*29)*		*Cocaine group (*N=*29)*		*Group comparison*	
	***Mean***	***s.d.***	***Mean***	***s.d.***	**t *or F***	**P**
Age (years)	36.8	±10.1	35.5	±8.7	0.53	0.599
Verbal IQ (NART score)	115.2	±5.9	104.1	±9.9	5.12	<0.001
Serum testosterone (nmol/L)^a^	15.4	±5.0	11.8	±5.1	2.70	0.009
Serum cortisol (nmol/L)^a^	282.3	±96.8	316.3	±146.0	−1.03	0.306
Testosterone–cortisol ratio	0.07	±0.04	0.04	±0.03	2.40	0.020
Dysphoric mood (BDI-II score)	3.3	±4.1	19.8	±12.0	−7.01	<0.001
State anxiety (STAI score)	28.8	±6.8	39.8	±14.4	−3.69	0.001
Trait anxiety (STAI score)	32.0	±8.5	47.2	±12.7	−5.29	<0.001
						
*Facial identity recognition (performance score)*						
Benton test	23.1	±1.9	22.6	±2.0	1.00	0.324
						
*Facial expression recognition accuracy (% correct responses)*						
Happy	97.8	±6.4	96.4	±7.5	0.57	0.455
Surprise	88.3	±13.3	87.9	±12.6	0.01	0.920
Fear	77.4	±15.5	60.9	±20.1	12.33	0.001
Sad	93.4	±12.3	89.1	±17.3	1.20	0.279
Disgust	72.9	±29.6	61.0	±32.8	2.10	0.152
Anger	80.0	±18.8	66.0	±24.2	6.03	0.017
						
*Facial expression recognition latency (mean response time in seconds)*						
Happy	1.45	±0.30	1.54	±0.61	0.55	0.462
Surprise	1.80	±0.51	2.03	±0.67	2.13	0.150
Fear	2.16	±0.84	2.29	±0.52	0.48	0.491
Sad	1.72	±0.49	1.91	±0.77	1.25	0.268
Disgust	1.97	±1.08	2.21	±1.09	0.73	0.398
Anger	2.14	±0.63	2.32	±0.66	1.13	0.292

Abbreviations: BDI-II, Beck Depression Inventory; NART, National Adult Reading Test; STAI, State–Trait Anxiety Inventory.

a Reference ranges provided by the laboratory: testosterone 8–29 nmo/L and cortisol 280–650 nmol/L.

**Table 2:** Summary of hierarchical regression analyses for variables predicting successful recognition of facial emotions and underneath the brain-behavior relationships (as reflected by the brain scores)

*Predicting variables*	*Fear recognition*			*Predicting variables*	*Fear recognition*		
	B	*SEB*	β		B	*SEB*	β
*Step 1*				*Step 2*			
Cocaine dependence	−14.61	6.25	−0.37*	Cocaine dependence	−7.77	7.48	−0.20
Opiate dependence	−11.68	6.87	−0.24	Opiate dependence	−10.81	6.47	−0.22
Alcohol dependence	9.06	7.28	0.17	Alcohol dependence	10.45	6.94	0.19
Δ*R*^2^			0.25**	Verbal IQ (NART)	0.85	0.29	0.42**
				Depressive mood (BDI-II)	−0.18	0.36	−0.11
				Anxiety (STAI-T)	0.33	0.30	0.22
				Δ*R*^2^			0.13*
							

*Predicting variables*	*Brain score fear*			*Predicting variables*	*Brain score fear*		
	B	*SEB*	β		B	*SEB*	β
*Step 1*				*Step 2*			
Cocaine dependence	−6.12	2.77	−0.35*	Cocaine dependence	−2.76	3.33	−0.16
Opiate dependence	−5.25	3.02	−0.25	Opiate dependence	−4.7	2.87	−0.22
Alcohol dependence	5.9	3.2	0.25	Alcohol dependence	6.87	3.07	0.29*
Δ*R*^2^			0.25**	Verbal IQ (NART)	0.31	0.13	0.35*
				Depressive mood (BDI-II)	−0.21	0.16	−0.30
				Anxiety (STAI-T)	0.21	0.13	0.32
				Δ*R*^2^			0.13*
							

*Predicting variables*	*Anger recognition*			*Predicting variables*	*Anger recognition*		
	B	*SEB*	β		B	*SEB*	β
*Step 1*				*Step 2*			
Cocaine dependence	−1.22	6.47	−0.03	Cocaine dependence	12.58	7.71	0.28
Opiate dependence	−32.94	7.1	−0.60**	Opiate dependence	−31.62	6.67	−0.58**
Alcohol dependence	1.46	7.53	0.02	Alcohol dependence	1.93	7.16	0.03
Δ*R*^2^			0.38**	Verbal IQ (NART)	0.81	0.3	0.35**
				Depressive mood (BDI-II)	−0.28	0.37	−0.15
				Anxiety (STAI-T)	−0.04	0.31	−0.02
				Δ*R*^2^			0.11*
							

*Predicting variables*	*Brain score anger*			*Predicting variables*	*Brain score anger*		
	B	*SEB*	β		B	*SEB*	β
*Step 1*				*Step 2*			
Cocaine dependence	−1.32	2.99	−0.07	Cocaine dependence	3.02	3.74	0.15
Opiate dependence	−12.13	3.27	−0.52**	Opiate dependence	−11.53	3.22	−0.49
Alcohol dependence	4.53	3.46	0.17	Alcohol dependence	5.24	3.46	0.20
Δ*R*^2^			0.30**	Verbal IQ (NART)	0.21	0.14	0.21*
				Depressive mood (BDI-II)	−0.24	0.18	−0.30
				Anxiety (STAI-T)	0.11	0.15	0.15
				Δ*R*^2^			0.07

Abbreviations: *B*, β-coefficient; *β*: standardized β-coefficient for the regression model; BDI-II, Beck Depression Inventory; NART, National Adult Reading Test; R^2^, coefficient of determination; SEB, standard error of the β-coefficient; STAI-T, State-Trait Anxiety Inventory (trait subscale); Δ, change.

Fear recognition: the significant effect of cocaine dependence at step 1 does not survive when verbal IQ is included in the model in step 2, suggesting that verbal IQ mediates fear recognition performance in cocaine-dependent individuals. Anger recognition: opiate dependence shows a highly significant effect at stage 1, and this effect survives the inclusion of verbal intelligence in the model. *P* is probability (*P<0.05; **P<0.001. The top half of the table shows the results for fear and the bottom half shows the results for anger.
