# Supplementary material for: In the face of threat: neural and endocrine correlates of impaired facial emotion recognition in cocaine dependence
Source: Transl Psychiatry. 2015 May 26;5(5):e570–. doi: 10.1038/tp.2015.58 (PMC4471289; doi:10.1038/tp.2015.58)
Supplement: Supplementary Information [file tp201558x1.doc]

Supplemental Material

In the face of threat: neural and endocrine correlates of impaired facial affect recognition in cocaine dependence

Ersche, K.D., Hagan, C.C., Smith D.G. Jones, P.S., Calder A.J.✝, and Williams, G.B.

✝ This paper is dedicated to Andy Calder, who sadly passed away during the preparation of this manuscript.

**Correspondence**:

Dr Karen Ersche, University of Cambridge, Department of Psychiatry, Herchel Smith Building for Brain & Mind Sciences, Cambridge Biomedical Campus, Cambridge CB2 0SZ, UK. Phone: +44 (0)1223 336587, Fax: +44 (0)1223 336581, e-mail: ke220@cam.ac.uk

**Table S1:** Brain regions ordered by decreasing magnitude of salience for fearful expression recognition. The locations of clusters are reported in MNI co-ordinates.

| **Anatomical Label** | **Cluster Size** | | **Voxel Mean** | **X (mm)** | | | **Y (mm)** | **Z (mm)** | **Salience** | **P** |
| --- | --- | --- | --- | --- | --- | --- | --- | --- | --- | --- |
| Precentral L | 3987 | | 0.40 | | 8 | | -38 | 58 | 8.54 | 0.0000 |
| Paracentral Lobule R | 567 | | 0.07 | | -16 | | -52 | -68 | 7.25 | 0.0000 |
| Cingulum Mid L | 3018 | | 0.18 | | 0 | | 62 | 12 | 6.47 | 0.0000 |
| Frontal Inf Orb R | 1157 | | 0.52 | | -54 | | 34 | 0 | 5.86 | 0.0000 |
| Thalamus L | 1248 | | 0.53 | | -60 | | -46 | 0 | 5.77 | 0.0000 |
| Frontal Mid R | 72 | | 0.33 | | -28 | | -2 | -50 | 5.51 | 0.0000 |
| Frontal Sup Medial R | 56 | | 0.59 | | 36 | | 6 | -20 | 5.37 | 0.0000 |
| Precentral L | 321 | | 0.11 | | -14 | | 4 | 26 | -5.34 | 0.0000 |
| Occipital Mid R | 34 | | 0.25 | | 54 | | -24 | 56 | 5.31 | 0.0000 |
| Occipital Mid L | 25 | | 0.17 | | -20 | | 44 | 22 | 5.12 | 0.0000 |
| Cerebelum R | 196 | | 0.79 | | 28 | | -18 | -14 | -5.04 | 0.0000 |
| Frontal Inf Tri R | 87 | | 0.10 | | -14 | | 28 | 2 | -5.00 | 0.0000 |
| Frontal Inf Tri L | 608 | | 0.38 | | 24 | | 20 | -30 | 4.81 | 0.0000 |
| Temporal Mid L | 250 | | 0.57 | | -8 | | 34 | 30 | 4.78 | 0.0000 |
| Frontal Sup Medial L | 164 | | 0.21 | | 50 | | 44 | 14 | 4.78 | 0.0000 |
| Parietal Inf R | 42 | | 0.26 | | 30 | | -26 | 12 | 4.76 | 0.0000 |
| Precentral L | 168 | | 0.16 | | -10 | | 26 | 22 | 4.73 | 0.0000 |
| Postcentral R | 994 | | 0.10 | | 58 | | -68 | 24 | 4.68 | 0.0000 |
| Postcentral R | 58 | | 0.53 | | 30 | | -54 | 40 | -4.68 | 0.0000 |
| Calcarine R | 87 | | 0.24 | | 32 | | -26 | 70 | 4.66 | 0.0000 |
| Pallidum R | 95 | | 0.49 | | -46 | | -82 | -12 | 4.60 | 0.0000 |
| Parietal Sup L | 60 | | 0.10 | | 32 | | -78 | 50 | 4.51 | 0.0000 |
| Temporal Inf L | 28 | | 0.19 | | 10 | | 2 | 36 | -4.49 | 0.0000 |
| Amygdala R | 33 | | 0.22 | | 18 | | -42 | 50 | -4.45 | 0.0000 |
| Temporal Pole Sup L | 51 | | 0.14 | | 24 | | 46 | -20 | 4.37 | 0.0000 |
| Temporal Pole Sup L | 99 | | 0.19 | | -20 | | -36 | 10 | 4.31 | 0.0000 |
| Pallidum L | 75 | | 0.46 | | 20 | | -72 | 46 | 4.30 | 0.0000 |
| Frontal Sup L | 36 | | 0.57 | | -22 | | 54 | 30 | 4.30 | 0.0000 |
| Cerebelum R | 34 | | 0.34 | | -12 | | 2 | 40 | -4.27 | 0.0000 |
| Frontal Sup R | 304 | | 0.25 | | 54 | | 2 | 26 | 4.25 | 0.0000 |
| Cingulum Post L | 30 | | 0.22 | | 28 | | 46 | 4 | 4.25 | 0.0000 |
| Temporal Pole Sup R | 188 | | 0.48 | | -6 | | -76 | -4 | 4.24 | 0.0000 |
| Occipital Mid L | 131 | | 0.68 | | -32 | | 4 | -18 | 4.23 | 0.0000 |
| Fusiform R | 110 | | 0.45 | | -46 | | 4 | -8 | -4.21 | 0.0000 |
| **Anatomical Label** | **Cluster Size** | | **Voxel Mean** | | **X (mm)** | | **Y (mm)** | **Z (mm)** | **Salience** | **P** |
| Vermis | 73 | | 0.76 | | 44 | | -60 | -16 | -4.21 | 0.0000 |
| Lingual L | 51 | | 0.14 | | -24 | | -76 | 6 | -4.20 | 0.0000 |
| Parietal Sup L | 22 | | 0.32 | | -52 | | -26 | 28 | -4.13 | 0.0000 |
| Precuneus L | 326 | | 0.1 | | 18 | | -48 | 10 | 4.05 | 0.0001 |
| Temporal Mid L | 133 | | 0.18 | | -8 | | -36 | 6 | 4.03 | 0.0001 |
| Occipital Inf R | 73 | | 0.41 | | -30 | | -36 | 66 | 4.00 | 0.0001 |
| Calcarine R | 35 | | 0.50 | | -54 | | 4 | 42 | 4.00 | 0.0001 |
| Temporal Sup L | 33 | | 0.07 | | 4 | | -48 | -28 | -4.00 | 0.0001 |
| Supp Motor Area L | 49 | | 0.54 | | -36 | | -68 | 48 | 3.99 | 0.0001 |
| Temporal Sup L | 77 | | 0.17 | | 36 | | -90 | -22 | 3.94 | 0.0001 |
| Temporal Sup R | 205 | | 0.56 | | -60 | | -2 | 30 | 3.91 | 0.0001 |
| Cerebelum Crus R | 32 | | 0.19 | | 4 | | -24 | 14 | -3.87 | 0.0001 |
| Cerebelum Crus L | 209 | | 0.82 | | -34 | | -22 | -14 | -3.86 | 0.0001 |
| Parietal Inf L | 66 | | 0.58 | | 60 | | 2 | -4 | 3.84 | 0.0001 |
| Parietal Sup R | 22 | | 0.36 | | 40 | | -26 | 46 | -3.81 | 0.0001 |
| Paracentral Lobule R | 20 | | 0.17 | | -16 | | -26 | 46 | 3.71 | 0.0002 |
| Temporal Mid L | 211 | | 0.4 | | -12 | | 52 | 32 | 3.70 | 0.0002 |
| Postcentral R | 24 | | 0.17 | | 32 | | 28 | 56 | -3.70 | 0.0002 |
| Parietal Sup R | 25 | | 0.6 | | -50 | | -40 | 52 | 3.69 | 0.0002 |
| Precentral R | 22 | | 0.3 | | -36 | | -66 | -8 | 3.68 | 0.0002 |
| Temporal Sup R | 23 | | 0.62 | | -18 | | 10 | 66 | 3.64 | 0.0003 |
| Cingulum Mid L | 26 | | 0.27 | | -32 | | 24 | -32 | 3.62 | 0.0003 |
| Postcentral L | 53 | | 0.35 | | -46 | | 4 | 18 | 3.59 | 0.0003 |
| Temporal Sup L | 68 | | 0.47 | | -18 | | 50 | -14 | 3.58 | 0.0003 |
| Cerebelum L | 116 | | 0.70 | | 14 | | 4 | 20 | -3.55 | 0.0004 |
| Vermis | 49 | | 0.63 | | -18 | | 20 | 60 | 3.52 | 0.0004 |
| Temporal Inf L | 95 | | 0.29 | | -22 | | 30 | -26 | 3.50 | 0.0005 |
| Temporal Pole Sup L | 70 | | 0.57 | | -18 | | -12 | 70 | 3.47 | 0.0005 |
| Frontal Sup R | 27 | | 0.21 | | 16 | | -72 | 60 | -3.47 | 0.0005 |
| Temporal Mid R | 119 | | 0.33 | | -46 | | -42 | -46 | 3.46 | 0.0005 |
| Frontal Mid L | 39 | | 0.04 | | 46 | | -74 | 46 | 3.46 | 0.0006 |
| Cerebelum L | 25 | | 0.51 | | -28 | | 52 | 28 | -3.43 | 0.0006 |
| Parietal Sup L | 34 | | 0.16 | | -10 | | -36 | 26 | -3.41 | 0.0007 |
| Cerebelum Crus R | 25 | | 0.19 | | 16 | | -86 | 44 | 3.37 | 0.0007 |
| Temporal Inf R | 32 | | 0.5 | | -32 | | -84 | -8 | 3.30 | 0.0010 |
| Cuneus R | 20 | | 0.38 | | 18 | | -72 | 12 | 3.29 | 0.0010 |
| Fusiform R | 48 | | 0.59 | | 48 | | -10 | -14 | 3.28 | 0.0010 |
| **Anatomical Label** | | **Cluster Size** | **Voxel Mean** | | **X (mm)** | **Y (mm)** | | **Z (mm)** | **Salience** | **P** |
| Fusiform L | 25 | | 0.57 | | 54 | | -56 | -36 | 3.21 | 0.0013 |
| Frontal Sup L | 21 | | 0.22 | | 4 | | -56 | -62 | 3.18 | 0.0015 |
| Frontal Sup Medial L | 22 | | 0.48 | | 12 | | 42 | 18 | 3.15 | 0.0016 |
| Temporal Inf R | 40 | | 0.39 | | -20 | | -60 | 0 | 3.14 | 0.0017 |
| Temporal Inf R | 20 | | 0.27 | | 10 | | -6 | 38 | 3.07 | 0.0022 |
| SupraMarginal R | 60 | | 0.48 | | 6 | | -86 | 14 | 3.00 | 0.0027 |
| Occipital Mid R | 31 | | 0.58 | | -6 | | 32 | -16 | 2.98 | 0.0029 |
| Cerebelum Crus R | 21 | | 0.15 | | 58 | | -70 | 4 | 2.96 | 0.0031 |
| Temporal Mid L | 35 | | 0.52 | | -38 | | 22 | 46 | 2.95 | 0.0032 |
| Hippocampus R | 21 | | 0.57 | | 54 | | 8 | 8 | 2.87 | 0.0041 |
| Hippocampus L | 28 | | 0.41 | | -4 | | -96 | 16 | 2.86 | 0.0043 |
| Vermis | 22 | | 0.16 | | 0 | | 2 | -28 | 2.83 | 0.0046 |
| Cerebelum Crus L | 36 | | 0.42 | | -56 | | -8 | 44 | 2.82 | 0.0049 |
| Frontal Sup R | 23 | | 0.11 | | -60 | | 24 | 18 | 2.69 | 0.0071 |
| Cerebelum Crus L | 26 | | 0.47 | | 30 | | -44 | 44 | 2.59 | 0.0097 |

**Table S2:** Brain regions ordered by decreasing magnitude of salience for angry facial
expression recognition. The locations of clusters are reported in MNI co-ordinates.

| **Anatomical Label** | **Cluster Size** | **Voxel Mean** | **X (mm)** | **Y (mm)** | **Z (mm)** | **Salience** | **P** |
| --- | --- | --- | --- | --- | --- | --- | --- |
| Precentral L | 4700 | 0.56 | -58 | 0 | 24 | 11.45 | 0.0000 |
| Paracentral Lobule R | 2930 | 0.42 | 10 | -40 | 58 | 8.64 | 0.0000 |
| Cingulum Mid L | 330 | 0.51 | -8 | -38 | 52 | 8.53 | 0.0000 |
| Frontal Inf Orb R | 501 | 0.68 | 34 | 32 | -16 | 8.15 | 0.0000 |
| Thalamus L | 114 | 0.14 | -20 | -28 | 2 | 7.92 | 0.0000 |
| Frontal Mid R | 2974 | 0.31 | 42 | 28 | 46 | 7.89 | 0.0000 |
| Frontal Sup Medial R | 3420 | 0.41 | 12 | 56 | 26 | 7.20 | 0.0000 |
| Occipital Mid R | 3105 | 0.36 | 42 | -78 | 36 | 6.64 | 0.0000 |
| Occipital Mid L | 1702 | 0.59 | -36 | -90 | 4 | 6.42 | 0.0000 |
| Frontal Inf Tri L | 995 | 0.58 | -52 | 36 | 0 | 6.25 | 0.0000 |
| Temporal Mid L | 133 | 0.54 | -54 | -4 | -24 | 6.00 | 0.0000 |
| Frontal Sup Medial L | 111 | 0.30 | -2 | 62 | 8 | 5.95 | 0.0000 |
| Parietal Inf R | 70 | 0.51 | 50 | -48 | 38 | 5.76 | 0.0000 |
| Precentral L | 71 | 0.37 | -36 | -18 | 62 | 5.75 | 0.0000 |
| Hippocampus R | 227 | 0.24 | 38 | -28 | -8 | -5.74 | 0.0000 |
| **Anatomical Label** | **Cluster Size** | **Voxel Mean** | **X (mm)** | **Y (mm)** | **Z (mm)** | **Salience** | **P** |
| Postcentral R | 72 | 0.34 | 14 | -34 | 76 | 5.71 | 0.0000 |
| Calcarine R | 316 | 0.10 | 18 | -48 | 10 | 5.67 | 0.0000 |
| Pallidum R | 30 | 0.01 | 14 | 0 | -2 | 5.61 | 0.0000 |
| Parietal Sup L | 42 | 0.48 | -20 | -54 | 66 | 5.54 | 0.0000 |
| Hippocampus L | 268 | 0.5 | -36 | -28 | -8 | -5.54 | 0.0000 |
| Temporal Pole Sup L | 75 | 0.33 | -28 | 22 | -32 | 5.53 | 0.0000 |
| Vermis | 30 | 0.12 | -4 | -52 | -28 | -5.06 | 0.0000 |
| Cerebelum Crus L | 145 | 0.18 | -26 | -90 | -38 | -5.01 | 0.0000 |
| Temporal Pole Sup L | 149 | 0.60 | -36 | 6 | -26 | 4.94 | 0.0000 |
| Pallidum L | 44 | 0.01 | -14 | 0 | -2 | 4.81 | 0.0000 |
| Frontal Sup L | 50 | 0.52 | -18 | 66 | 8 | 4.80 | 0.0000 |
| Frontal Sup R | 54 | 0.21 | 18 | 8 | 52 | 4.75 | 0.0000 |
| Cingulum Post L | 189 | 0.1 | -16 | -46 | 8 | 4.71 | 0.0000 |
| Temporal Pole Sup R | 244 | 0.19 | 54 | 18 | -8 | 4.67 | 0.0000 |
| Occipital Mid L | 83 | 0.52 | -40 | -72 | 20 | 4.63 | 0.0000 |
| Frontal Sup R | 39 | 0.42 | 24 | 16 | 64 | -4.61 | 0.0000 |
| Precuneus L | 36 | 0.37 | -14 | -52 | 34 | 4.57 | 0.0000 |
| Temporal Mid L | 36 | 0.22 | -46 | -50 | -4 | 4.53 | 0.0000 |
| Occipital Inf R | 339 | 0.54 | 30 | -88 | -12 | 4.46 | 0.0000 |
| Cerebelum Crus L | 183 | 0.31 | -32 | -88 | -30 | -4.46 | 0.0000 |
| Calcarine R | 149 | 0.33 | 12 | -84 | 16 | 4.26 | 0.0000 |
| Supp Motor Area L | 242 | 0.40 | 0 | 16 | 48 | 4.24 | 0.0000 |
| Temporal Sup L | 88 | 0.49 | -54 | -2 | -8 | 4.21 | 0.0000 |
| Temporal Sup R | 56 | 0.51 | 58 | -6 | -2 | 4.12 | 0.0000 |
| Parietal Inf L | 84 | 0.49 | -56 | -26 | 50 | 4.11 | 0.0000 |
| Precentral L | 50 | 0.54 | -30 | -10 | 62 | -3.95 | 0.0001 |
| Paracentral Lobule R | 41 | 0.27 | 10 | -28 | 64 | 3.94 | 0.0001 |
| Cerebelum R | 281 | 0.65 | 26 | -36 | -28 | -3.88 | 0.0001 |
| Precentral R | 33 | 0.32 | 34 | -28 | 68 | 3.86 | 0.0001 |
| Temporal Mid L | 28 | 0.08 | -66 | -56 | -12 | 3.86 | 0.0001 |
| Frontal Inf Tri R | 23 | 0.53 | 52 | 38 | -2 | -3.83 | 0.0001 |
| Temporal Sup R | 39 | 0.39 | 50 | -36 | 14 | 3.77 | 0.0002 |
| Postcentral R | 31 | 0.42 | 48 | -34 | 56 | -3.76 | 0.0002 |
| Cingulum Mid L | 26 | 0.29 | -14 | -40 | 40 | 3.75 | 0.0002 |
| Postcentral L | 145 | 0.43 | -34 | -38 | 62 | 3.74 | 0.0002 |
| Temporal Sup L | 49 | 0.34 | -64 | -6 | 4 | 3.68 | 0.0002 |
| Vermis | 31 | 0.24 | 6 | -44 | 0 | 3.67 | 0.0002 |
| **Anatomical Label** | **Cluster Size** | **Voxel Mean** | **X (mm)** | **Y (mm)** | **Z (mm)** | **Salience** | **P** |
| Temporal Inf L | 251 | 0.35 | -50 | -40 | -14 | -3.67 | 0.0002 |
| Temporal Inf L | 139 | 0.14 | -68 | -34 | -22 | 3.63 | 0.0003 |
| Temporal Pole Sup L | 22 | 0.51 | -22 | 8 | -30 | 3.60 | 0.0003 |
| Amygdala R | 116 | 0.92 | 24 | -4 | -18 | -3.54 | 0.0004 |
| Temporal Mid R | 39 | 0.72 | 50 | -6 | -16 | 3.51 | 0.0005 |
| Frontal Mid L | 33 | 0.55 | -38 | 22 | 48 | 3.46 | 0.0005 |
| Cerebelum Crus R | 99 | 0.58 | 46 | -44 | -42 | 3.44 | 0.0006 |
| Temporal Inf R | 39 | 0.54 | 60 | -24 | -28 | 3.42 | 0.0006 |
| Cerebelum R | 43 | 0.48 | 16 | -44 | -46 | -3.42 | 0.0006 |
| Cuneus R | 21 | 0.43 | 16 | -82 | 42 | 3.36 | 0.0008 |
| Fusiform R | 34 | 0.81 | 40 | -56 | -18 | -3.36 | 0.0008 |
| Fusiform R | 55 | 0.36 | 26 | -78 | -4 | 3.35 | 0.0008 |
| Vermis | 49 | 0.50 | 0 | -42 | -16 | -3.33 | 0.0009 |
| Fusiform L | 24 | 0.54 | -32 | -48 | -10 | 3.26 | 0.0011 |
| Lingual L | 104 | 0.41 | -6 | -94 | -16 | -3.22 | 0.0013 |
| Parietal Sup L | 34 | 0.52 | -18 | -62 | 64 | -3.22 | 0.0013 |
| Temporal Sup L | 33 | 0.40 | -44 | -30 | 6 | -3.20 | 0.0014 |
| Cerebelum Crus R | 42 | 0.92 | 32 | -70 | -36 | -3.19 | 0.0014 |
| Frontal Sup L | 23 | 0.6 | -18 | 8 | 66 | 3.17 | 0.0015 |
| Cerebelum Crus L | 29 | 0.56 | -44 | -72 | -46 | -3.17 | 0.0015 |
| Frontal Sup Medial L | 35 | 0.47 | 2 | 34 | 40 | 3.16 | 0.0016 |
| Parietal Sup R | 30 | 0.38 | 20 | -70 | 58 | -3.15 | 0.0016 |
| Temporal Inf R | 58 | 0.76 | 58 | -46 | -10 | 3.11 | 0.0019 |
| Temporal Inf R | 21 | 0.65 | 50 | -70 | -4 | 3.11 | 0.0019 |
| Postcentral R | 30 | 0.40 | 24 | -30 | 58 | -3.03 | 0.0025 |
| SupraMarginal R | 36 | 0.37 | 50 | -36 | 32 | 2.95 | 0.0031 |
| Occipital Mid R | 30 | 0.14 | 30 | -98 | 14 | 2.93 | 0.0034 |
| Parietal Sup R | 24 | 0.14 | 38 | -56 | 62 | -2.92 | 0.0035 |
| Cerebelum L | 22 | 0.12 | -6 | -36 | -10 | -2.87 | 0.0041 |
| Frontal Sup R | 39 | 0.56 | 24 | -12 | 64 | -2.84 | 0.0045 |
| Cerebelum L | 34 | 0.27 | -18 | -38 | -52 | -2.80 | 0.0051 |
| Cerebelum Crus1 R | 21 | 0.41 | 38 | -56 | -36 | 2.74 | 0.0061 |
| Parietal Sup L | 22 | 0.07 | -38 | -62 | 60 | -2.74 | 0.0062 |
| Temporal Mid L | 24 | 0.46 | -46 | -32 | 2 | 2.73 | 0.0063 |
